# A Reference Finding Rarely Seen in Primary Hyperparathyroidism: Brown Tumor

**DOI:** 10.1155/2012/432676

**Published:** 2012-11-21

**Authors:** F. Mantar, S. Gunduz, U. R. Gunduz

**Affiliations:** ^1^Deparment of Endocrinology, Faculty of Health Sciences, Bahçeşehir University, 34353 Istanbul, Turkey; ^2^Department of Medical Oncology, Faculty of Medicine, Akdeniz University, 07070 Antalya, Turkey; ^3^Department of Surgery, Antalya Education Research Hospital, 07050 Antalya, Turkey

## Abstract

Primary hyperparathyroidism is an endocrinopathy which is characterized with the hypersecretion of parathormone. During the progress of the disease bone loss takes place due to resorption on the subperiosteal and endosteal surfaces. Brown tumor is a localized form of osteitis fibrosa cystica, being part of the hyperparathyroid bone disease. It is rarely the first symptom of hyperparathyroidism. Nowadays, the diagnosis is made at an asymptomatic or minimally symptomatic stage. We present a male patient presented with a massive painless swelling in the left maxilla as the first manifestation of primary hyperparathyroidism due to a parathyroid adenoma. Parathyroidectomy was performed, and there was a regression of the bone lesion, without the need of performing other local surgical procedures.

## 1. Introduction

The most common cause of hypercalcemia is hyperparathyroidism (PHPT) in outpatients. Incidence of PHPT is 2-3/1000 in women and 1/1000 in men. The disease is most frequent in postmenopausal women during the sixth decade of life, although it is seen in all age groups. Of patients with PHPT, 80–85% develop the disease due to parathyroid adenoma, 15–20% due to hyperplasia of the parathyroid gland, while in less than 0.5%, the cause is parathyroid carcinoma. Brown tumor is a reactive lesion in the bone tissue, very rarely seen during the course of PHPT and is secondary to localized rapid osteoclastic bone turnover resulting from the direct effect of parathormone. Hemorrhage, vascular fibrous tissue, and resultant granulation tissue replace normal bone marrow elements. Localized accumulation of fibrous tissues and giant cells results in bone expansion. These are reversible tissues with excision of the parathyroid adenoma or removal of the four glands [1–4].

## 2. Case Report

A 23-year-old male patient with no known additional disease history has noticed about 10 months ago a painless swelling first on the left side of his face which gradually generalized and presented to the dental clinic. The patient was treated with antibiotics for a while. When the swelling in the patient's face persisted despite the treatment, biopsy was performed to the affected part of the face. The patient was referred to our hospital for advanced examinations after the pathologic analyses demonstrated giant-cell reparative granuloma. 

**Figure 1:**
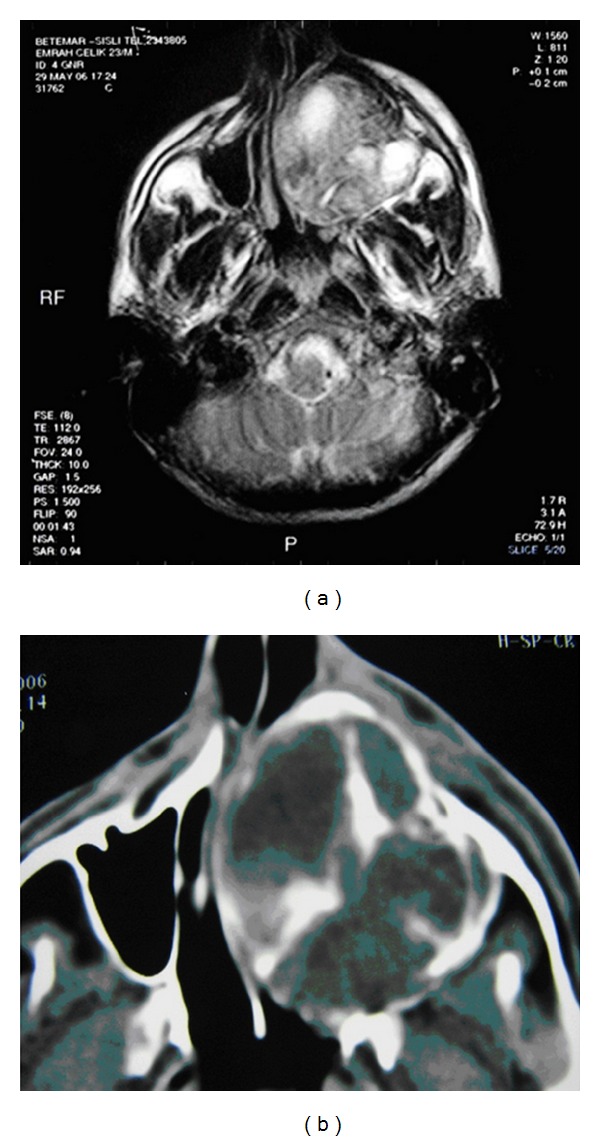
Cranial MR: multicentric osseous lesions in left maxillofacial bones.

**Figure 2:**
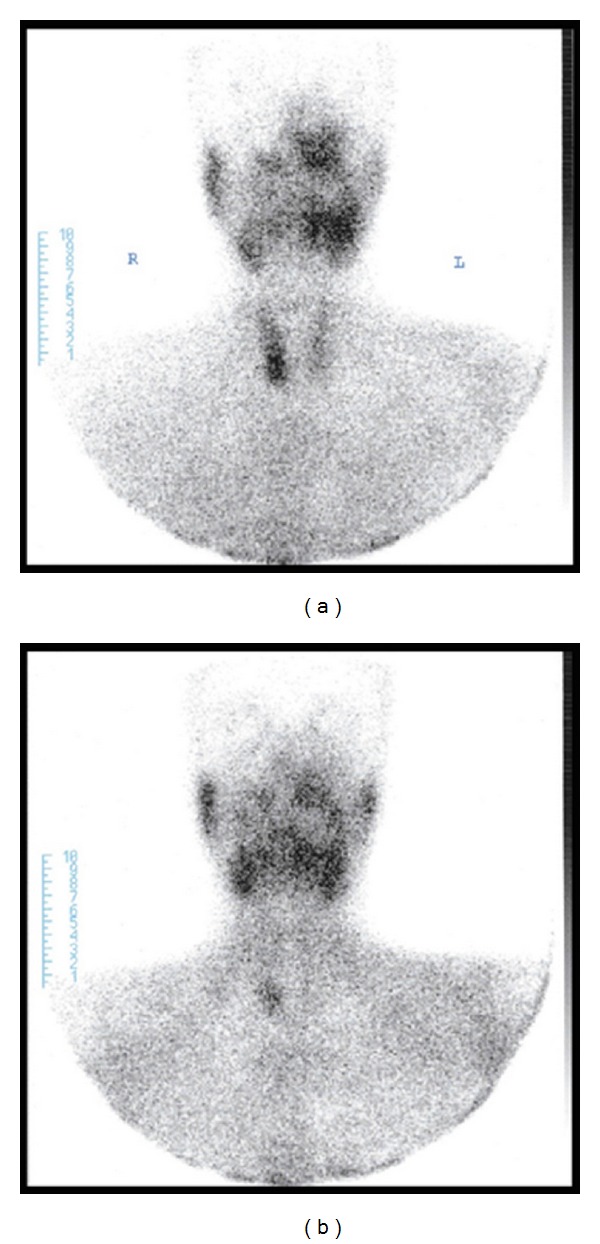
Sestamibi scintigraphy: parathyroid adenoma.

Physical examination of the patient revealed no additional pathologies other than the swelling in the left maxillary region. Upon noticing considerably increased levels of alkaline phosphatase and blood calcium and considerably decreased levels of blood phosphor (calcium: 14.6 mg/dL, phosphor: 2 mg/dL, alkaline phosphatase: 702 U/L (70–290)) in the results of the requested analyses, the patient was hospitalized at our clinic for emergency hypercalcemia treatment and advanced analyses. The patient was initiated on isotonic 5 L/day and furosemide 6 × 20 infusion for emergency treatment. Zoledronic acid 4 mg vial infusion was started as the calcium values did not decrease despite the treatment. With this treatment, patient's blood calcium levels returned to normal levels (Ca: 9.5 mg/dL) on the second day of the treatment. Because primary hyperparathyroidism was considered as the diagnosis of the patient when initiating the emergency treatment, intact parathormone (IPTH) and 24-hour urine calcium levels were requested from the laboratory. The results were indeed significantly high, supportive of our prediagnosis; IPTH: 1302 pg/mL (15–65) and 24-hour urine calcium: 617.5 mg/24 h. Although our diagnosis was confirmed biochemically, neck ultrasound and parathyroid scintigraphy (sestamibi scintigraphy) were requested to localize the adenoma in the patient. In addition, cranial MRI including the face was scheduled to fully understand the facial lesion radiologically and to be able to follow up after the planned surgery. The neck ultrasound revealed a 25 × 22 mm lesion containing a 10 × 5 mm hypoechoic space in the thyroid left lobe inferior. Expansile, remodeling, and multicentric osseous lesions of multilocular septate appearance localized at bilateral maxillary and left ramus-corpus mandibula, the largest of which was localized in left maxillofacial bones, were detected on cranial MRI analysis (Figure 1). Parathyroid sestamibi scintigraphy results were consistent with parathyroid adenoma (Figure 2). These findings confirmed the primary hyperparathyroidism diagnosis of the patient. The patient, after achieving reductions in blood calcium with emergency treatment, was referred to surgery. Total excision of the parathyroid adenoma guided by gamma probe was performed in the patient. The lesion in the maxillofacial region was judged to be Brown tumor secondary to primary hyperparathyroidism, based on both biopsy and radiographic findings. This lesion was monitored without any intervention. Presence of Brown tumor in other bones was investigated through long bone graphy and extremities and cranial direct graphies. No foci suggestive of another Brown tumor were identified. The pathology report was also consistent with parathyroid adenoma. Hungry bone syndrome developed following the surgery: PTH < 3 pg/mL and Ca: 8.2 mg/dL. The patient was administrated i.v. calcium treatment for two days and was initiated on calcitriol 0.5 tbl. 2 × 1 calcium tbl. 3 × 1. 

**Figure 3:**
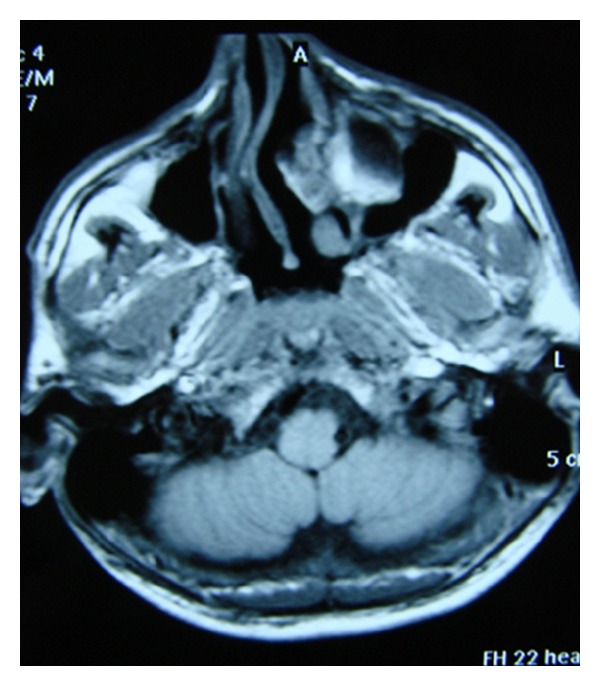
Cranial MR: regression of the Brown tumor which localized in the left maxillary region.

Regression in the Brown tumor localized in the left maxillary region was observed during followup (Figure 3). The patient is still on routine followup at our endocrinology clinic with normal levels of blood calcium. 

## 3. Discussion

Primary hyperparathyroidism is the third most common endocrinologic condition following diabetes mellitus and thyroid diseases. Its annual incidence has been reported as 28/100.000 in the USA [5]. Improper or excessive secretion of PTH results in PHPT. Of patients with PHPT, 80–85% develop the disease due to parathyroid adenoma, 15–20% due to hyperplasia of the parathyroid gland, while in less than 0.5% the cause is parathyroid carcinoma [6]. A single adenoma indicates sporadic disease, while hyperplasia of four glands suggests familial diseases (MEN type 1 or 2A). Its diagnosis is usually based on serum calcium and PTH levels higher than normal limits. In some cases, blood calcium levels within normal limits may also be observed. Today, patients with PHPT can be diagnosed in the early and asymptomatic period owing to the advances in the blood analysis methods [7]. 

Although many systems are affected in PHPT, the most pronounced alternations are observed in the bone. Approximately 2% of the patients are reported to develop osteitis fibrosa cystica, and 0.8% develop Brown tumor. These figures suggest a very low frequency of Brown tumors in these patients [8, 9]. Brown tumor is the localized form of osteitis fibrosa cystica [10] and is a reactive lesion in the bone tissue, very rarely seen during the course of PHPT. The tumor is secondary to localized rapid osteoclastic bone turnover resulting from the direct effect of PTH. Hemorrhage, vascular fibrous tissue, and resultant granulation tissue replace normal bone marrow elements. Localized accumulation of fibrous tissues and giant cells results in bone expansion [3]. Brown tumors are primarily observed in face, mandible and neck region, followed by the pelvis, ribs, and femur. Axial skeleton is rarely involved. They may occasionally cause pathologic fractures [9, 11]. 

Primary treatment of Brown tumors is surgical removal of associated parathyroid pathology. Brown tumors generally regress spontaneously over time [10]. Return of serum PTH, calcium, and phosphor levels to normal limits is an indicator of the success of treatment. Increases in bone mineral mass during the consequent period may also represent a significant indicator [12]. 

The patient may first present with the complaint of painless mass in the maxillary region, as was the case in our patient. There are very few cases in the literature describing Brown tumor localized in the maxillary and facial regions in primary hyperparathyroidism. Diagnosis may be easily overlooked in such cases. Therefore, we must consider primary hyperparathyroidism in differential diagnosis by analyzing blood calcium and PTH levels of patients presenting with mass in bones, particularly in the face. Establishing an accurate diagnosis will eliminate patient's problem through the surgery of the parathyroid gland and will avoid more advanced surgical interventions. It should be kept in mind that there may be a regression in the Brown tumor over years, as is the case in our patient.
